# UTRme: A Scoring-Based Tool to Annotate Untranslated Regions in Trypanosomatid Genomes

**DOI:** 10.3389/fgene.2018.00671

**Published:** 2018-12-18

**Authors:** Santiago Radío, Rafael Sebastián Fort, Beatriz Garat, José Sotelo-Silveira, Pablo Smircich

**Affiliations:** ^1^Department of Genomics, Instituto de Investigaciones Biológicas Clemente Estable, MEC, Montevideo, Uruguay; ^2^Laboratory of Molecular Interactions, Facultad de Ciencias. Universidad de la República, Montevideo, Uruguay; ^3^Department of Cell and Molecular Biology, Facultad de Ciencias, Universidad de la República, Montevideo, Uruguay

**Keywords:** post transcriptional regulation, untranslated region, UTR prediction software, prediction score, GUI

## Abstract

Most signals involved in post-transcriptional regulatory networks are located in the untranslated regions (UTRs) of the mRNAs. Therefore, to deepen our understanding of gene expression regulation, delimitation of these regions with high accuracy is needed. The trypanosomatid lineage includes a variety of parasitic protozoans causing a significant worldwide burden on human health. Given their peculiar mechanisms of gene expression, these organisms depend on post-transcriptional regulation as the main level of gene expression control. In this context, the definition of the UTR regions becomes of key importance. We have developed UTR-mini-exon (UTRme), a graphical user interface (GUI) stand-alone application to identify and annotate 5′ and 3′ UTR regions in a highly accurate way. UTRme implements a multiple scoring system tailored to address the issue of false positive UTR assignment that frequently arise because of the characteristics of the intergenic regions. Even though it was developed for trypanosomatids, the tool can be used to predict 3′ sites in any eukaryote and 5′ UTRs in any organism where trans-splicing occurs (such as the model organism *C. elegans*). UTRme offers a way for non-bioinformaticians to precisely determine UTRs from transcriptomic data. The tool is freely available via the conda and github repositories.

## Introduction

Post-transcriptional regulation is a key step to control gene expression levels in eukaryotes (Franks et al., 2017) that depends on factors recognizing signals mostly present in the UTRs of the mRNAs. These mechanisms are crucial in trypanosomatids since they lack transcription initiation control. The trypanosomatid lineage includes a variety of parasitic protozoans causing significant worldwide burden on human health (Prüss-Ustün et al., 2016). Trypanosomatids represent early divergent eukaryotes that have evolved distinctive biological features; one of the most intriguing characteristic is the apparent lack of transcription initiation control, being initiation sites characterized only by chromatin modifications and DNA structural signals (Respuela et al., 2008; Siegel et al., 2009; Thomas et al., 2009; Wright et al., 2010; Ekanayake and Sabatini, 2011; Smircich et al., 2013; Ramos et al., 2015). This implies that the gene expression patterns result mainly from post-transcriptional control. Therefore, the regulation of mRNA localization (Pastro et al., 2017), stability (Fadda et al., 2014), and translatability (Jensen et al., 2014; Vasquez et al., 2014; Smircich et al., 2015) are key mechanisms to determine protein concentration. These processes depend on regulatory proteins which interact with RNA by recognizing either sequence or structural signals present mainly on the UTRs of the mRNAs (Clayton, 2013; De Gaudenzi et al., 2013; Pastro et al., 2013). So, to deepen our understanding of gene expression regulation and the involved signals we need to delimit these regions with high accuracy. The annotation of UTR regions has been a challenging task depending on specific experiments designed for each particular gene. However, transcriptomic approaches currently give the opportunity to annotate these sites on a global scale. Efforts have been carried out to provide tools that allow the definition of UTR boundaries in trypanosomatids (Fiebig et al., 2014; Dillon et al., 2015). Although these tools have proven useful (Dillon et al., 2015; Pastro et al., 2017), both the repetitive nature of the trypanosomatid genomes and the high abundance of poly(A) tracts present in their intergenic regions confound the algorithms. Therefore, we have developed UTRme (UTR-mini-exon), a stand-alone application to identify and annotate 5′ and 3′ UTR regions, implementing a multiple scoring system that addresses both the aforementioned and several other issues that arise during the UTR annotation process. The tool provides not only the annotation but also a score that enables to discriminate the certainty of that annotation improving the usability of the results. Additionally, UTRme offers a Graphical User Interface (GUI) which turns it user friendly to non-bioinformaticians and, as a stand-alone application, can be scaled to any project depending only on the user's hardware. UTRme reports annotation and sequence files and plots general characteristics of the resulting data (such as the distribution of UTR lengths, UTRme scores and number of processing sites per gene). The 5′ UTR prediction can be easily extended to any organism where trans-splicing occurs, like the model organism *C. elegans*, among others (Lei et al., 2016). Furthermore, UTRme can be used for 3′ UTR prediction in any eukaryote. The source code is freely available at https://github.com/sradiouy/UTRme and can be easily installed via the conda repository on a linux based systems with a single command “*conda install -c sradiouy utrme*.”

## Methods

### Genome Data

Genomic and coding sequences (cds) annotation files where downloaded from TritrypDB (http://tritrypdb.org/) release 35.

### Transcriptomic Data Simulation

In order to test the software accuracy, a 30x 100 bp pair-end RNA-seq run was simulated using the Piquant package (https://github.com/lweasel/piquant). This package simulates sequencing errors and platform bias. To simulate reads originating from full transcripts [including UTRs, SL, and poly(A) sequences] a random length UTR was added to each *T. cruzi* coding sequence. For 5′ UTRs a maximum length of 101 bp was allowed while for 3′ UTRS the maximum length was set to 301 pb. The SL sequence or a 35 pb poly(A) tail was added to each end accordingly.

### 5′ End Enriched RNA-seq Library Construction

First strand of cDNA was prepared with 3 μg of purified RNA, random hexamers and Invitrogen SuperScript® III First-Strand Synthesis System (Pub. No. MAN0001346). Second strand of cDNA was prepared using a specific SL primer (5′tacagtttctgtactatattg3′) and DNA Polymerase I Large (Klenow) Fragment (NEB M0210). Library preparation protocol included end-repair, adapters ligation, size selection (Pipping Prep SAGE System), and amplification of the library using manufacturer's recommended protocol Ion plus fragment library kit (Pub. No. MAN0009847). Qualitative and quantitative assessment of the libraries was analyzed by Agilent 2100 Bioanalyzer System, using HS DNA 1000 reagents (Agilent Technologies). Emulsion amplification of the library was performed using Ion Onetouch 2 System with the Ion PGM Template OT2 Hi-Q view 400 kit (Pub. No. MAN0014579). Ion Sphere Particles (ISPs) enrichment step was performed on the Ion OneTouch ES system (Pub. No. MAN0014579). The Ion PGM system was used for sequencing using Ion PGM Hi-Q view Sequencing Solutions and Ion 318 Chip v2, following the manufacturer's recommended protocol for 400 bp reads (Pub. No. MAN0014583). (SRA BioProject PRJNA473354).

## Results and Discussion

### Pipeline Description

**Figure 1:**
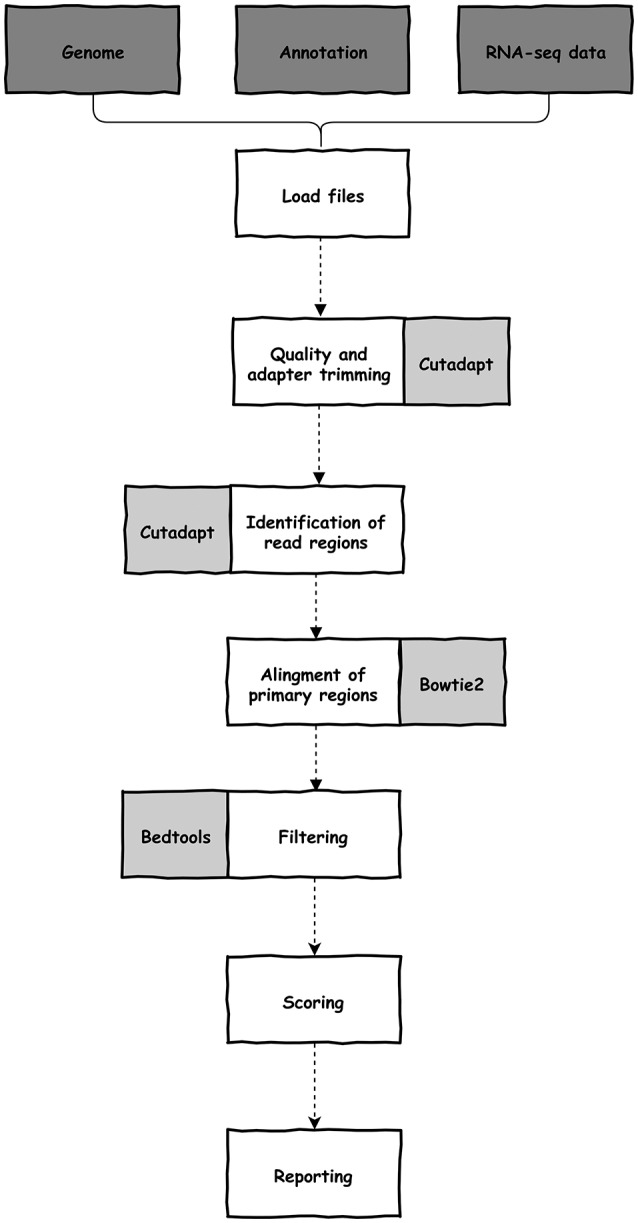
Outline of the UTRme pipeline. Required initial files, data processing steps and software packages used during processing are depicted in dark gray, white and light gray backgrounds, respectively.

The software was written in python (version 3) and depends on cutadapt (Martin, 2011), bedtools (Quinlan and Hall, 2010), bowtie2 (Langmead and Salzberg, 2012), samtools (Li et al., 2009), and python and unix modules. All dependencies are automatically configured during installation. UTRme needs a reference genome (sequence and cds annotation) and raw reads from an RNA-seq experiment (single-end or paired-end) (Figure 1). These required files, and optional arguments are selected through the GUI. Documentation, including a preview of the GUI, is available at https://github.com/sradiouy/UTRme.

**Figure 2:**
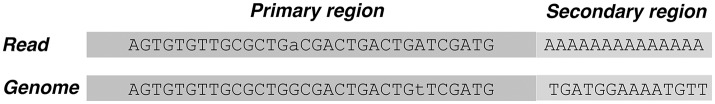
UTRme classification of read regions. Regions of each read and their counterparts in the genome are defined by UTRme as primary and secondary.

The pipeline starts with the removal of adapter sequences and trimming of low-quality ends from reads using cutadapt. By default, UTRme trims the Illumina TrueSeq adapter, but any sequence can be specified. Afterwards, the trimming software is also used to identify and clip the reads containing putative poly(A) tails or spliced leader (SL) sequences, allowing for mismatches. By default, an error probability 0.01 for poly(A) sequences (adjustable by the user) and one mismatch for SL sequences are defined. To correctly identify the trans-splicing sites, the organism must be specified. Currently, *Leishmania major, Trypanosoma brucei*, and *Trypanosoma cruzi* are available, however other species can be included by adding specific SL sequences. This trimming process allows us to define two regions on a read (Figure 2). The primary region is the sequence that was left after read trimming, while the secondary region is the putative poly(A) tail or SL sequence recognized by cutadapt.

The primary regions of the reads are aligned to the genome using bowtie2 applying the default very-sensitive local end-to-end alignment mode (Figure 1). The subset of reads aligning to intergenic regions is selected using bedtools. The mapping of the primary regions defines the putative splice acceptor site or poly(A) addition sites. At this point, UTRme evaluates in detail each putative site to assess its reliability by reporting a score that quantitates the confidence of the UTR site definition. This metric is calculated by combining an individual score that indicates the confidence with which each read predicts a given site, and global score that considers the cumulative evidence of all the reads that support a single processing site (see Supplementary File 1 for a detailed description of all the scores and their calculation). The individual score includes three components: the primary, secondary and accessory scores. As read mapping is not always accurate, the primary score aims to assess the likelihood that the primary region was indeed transcribed in the genomic region that it was mapped to. This is estimated based on the evaluation of their similarity using a modified version of the Damerau-Levenshtein algorithm (Levenshtein, 1966; Majorek et al., 2014) implemented in the fuzzywuzzy python library (https://github.com/seatgeek/fuzzywuzzy). This metric evaluates the minimum number of changes that are required to go from string A to string B considering mismatches and gaps. Once the primary score has been measured and the read is not discarded, the secondary score is calculated. This evaluates the difference between the secondary region [putative poly (A) tail or SL sequence] and the genomic region contiguous to the primary region [by calculating the Hamming distance; (He et al., 2004)]. A true processing event would result in a sequence that is independent of this genomic region, so the greater the difference between the secondary region and the genomic region, the higher the score. In trypansomatids, where a high number A tracts repeats are present in the intergenic regions (Duhagon et al., 2011), a poly(A) in a read could be the result of transcription and not mRNA processing. Another aspect to consider is the length of the secondary region. The longer this sequence, more likely it represents a true post transcriptional event and this is included in the score. Also, the number of adenines in the secondary genomic region is also considered; a higher proportion of As result in a smaller the score. Finally, UTRme also considers aspects that influence the reliability of the processing site determination (see Supplementary File 1). Most are used to fine tune the final individual score and depends on features such as the confidence that the read was not misplaced during mapping, the presence of specific splicing signals (AG acceptor and polypyrimidine tract [poly(Y)] and the existence of unannotated open reading frames (ORFs) or undetermined nucleotides (Ns) in the defined region. As an example, the presence and characteristics of a poly(Y) tracts upstream of the trans-splicing site is verified. We defined poly(Y) tracts as the longest tract of pyrimidines not interrupted by more than a single purine (Dillon et al., 2015). The presence and composition of the tract is analyzed, and scores are assigned considering their accordance with poly(Y) tract characteristics defined in (Siegel et al., 2005).

The global score considers the cumulative evidence of all the reads that support a single processing site giving a broader view of the accuracy of the site. For SL sites, it is proportional to the number of reads that support the site (“occurrences”). For poly(A) sites, in addition to the previous metric, the sequences of the putative poly(A) tail of all the reads that support the site is analyzed (for details see Supplementary File 1).

Finally, the reported score is calculated by adding the global score to the value of the third quartile of the individual's scores of all the reads that support that site. The maximum value for this score is set to 100. The higher the score the more confident is the prediction. All sites with positive scores are reported as they are supported by a reasonable amount of evidence. By default, if a site has a negative score it is not reported (this can be modified by the user).

In summary, the reported score recaps many aspects that influence the certainty that a site can be defined with the provided RNA-seq data.

### Assessment of UTRme Accuracy

UTRme takes about 1 h to process 90M paired reads in a middle-sized hardware configuration (40 cores−3 Gb max. RAM footprint). The results are presented as tab—delimited text or excel files, report plots, annotation and sequence files.

Tables include a full report that details both the basic information of the site (such as associated gene, UTR length, acceptor dinucleotide for the SL, and site score) and also the different computed scores and other features of the site (information about the poly(Y) tract for the SL, maximum ORF sequence in the UTR -if its length is greater than 30 amino acids-, among others) (Supplementary Table 1). A summary report is also created where only basic information for the best scoring site is informed for each gene (Table 1).

**Table 1:** Example of UTRme summary report output.

**Gene**	**utr_len**	**acceptor**	**score**	**occurrences**	**# sites**
TcCLB.397937.5	15	AG	89	418	4
TcCLB.398343.9	80	AG	79	2	2
TcCLB.399033.19	21	AG	90	27	4
TcCLB.400945.10	100	AG	85	39	4
TcCLB.404001.10	14	AG	95	59	3
TcCLB.404001.4	11	AG	91	75	5
TcCLB.404843.20	143	AG	92	65	2
TcCLB.405165.19	41	AG	92	54	4
TcCLB.407477.20	10	AG	91	64	2
TcCLB.407477.30	63	AG	96	51	4

*Summary report of best scoring epimastigote's SL sites using epimastigote RNA-seq data from Li et al. (2016). The first 10 lines are shown*.

**Figure 3:**
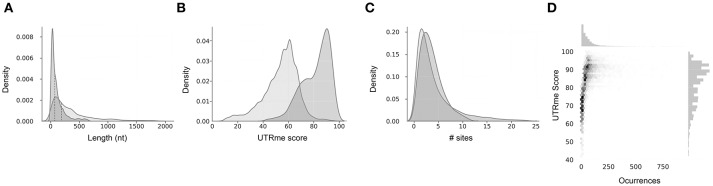
Example of UTRme summary plots output. Reported plots for the 5′ and 3′ UTRs predicted using *T. cruzi* epimastigote Y strain RNA-seq data from Li et al. (2016). Plots for 5′ and 3′ UTRs are in dark gray and light gray, respectively. **(A)** Kernel density estimation plot of UTR lengths. **(B)** Kernel density estimation plot of both 5′ and 3′ UTR score distribution. **(C)** Kernel density estimation plot for the number of 5′ and 3′ UTR sites. In all cases the median is indicated as a dotted line. **(D)** Central panel: Scatter plot of 5′ UTR scores vs occurrences. A higher point density is indicated by a darker color for each bin. Upper panel: histogram of occurrences. Right panel: histogram of scores.

UTRme generates both a sequence fasta file containing the sequences of the UTRs, as well as an annotation gff file that allows visualization and further analysis (Supplementary Figure 1). This output is provided for all the sites and for the best scoring sites separately. Finally, the reported plots show general properties of the predicted UTRs (UTR lengths, scores, occurrences vs scores, number of sites per gene) (Figure 3).

To test the accuracy of the software, RNA-seq data from *T. cruzi* epimastigotes was obtained using an approach aimed to obtain a 5′ end enriched library. To improve mapping accuracy the average read size was set to 400 nt (see Methods section). 5′ processing sites were defined using UTRme and the best scoring ones where checked against previously published UTRs that were described though specific experimental approaches (Table 2) (Bontempi et al., 1994; Di Noia et al., 1998, 2000; Vandersall-Nairn et al., 1998; Teixeira et al., 1999; Búa et al., 2001; D'Orso and Frasch, 2001; Bartholomeu et al., 2002; Bhatia et al., 2004; Coelho et al., 2006; García et al., 2010).

**Table 2:** Comparison of UTRme predictions against experimentally defined processing sites.

**Site**	**Gene**	**UTRme 5^**′**^ enriched**	**UTRme Li**	**UTRme Pastro**	**SlaP mapper pastro**	**Exp**.	**Article**
5′	TcCLB.509147.50	48	51	51	54	55	Di Noia et al., 2000
5′	TcCLB.511679.10	51	51	51	54	51	Di Noia et al., 2000
3′	TcCLB.506533.142	786	786	764	–	789	Di Noia et al., 2000
3′	TcCLB.511679.10	–	375	–	–	~353	Di Noia et al., 2000
5′	TcCLB.507485.140	–	140	137	–	137	Teixeira et al., 1999
5′	TcCLB.506407.10	93	102	101	718	103	Vandersall-Nairn et al., 1998
5′	TcCLB.509123.10	–	33	–	–	33	García et al., 2010
5′	TcCLB.505931.50	43	76	72	43	76	Bontempi et al., 1994
5′	TcCLB.507093.220	68	66	68	–	68	D'Orso and Frasch, 2001
5′	TcCLB.507639.30	42	42	42	42	42	Coelho et al., 2006
5′	TcCLB.507511.81	–	41	41	–	41	Di Noia et al., 1998
5′	TcCLB.510241.70	–	144	144	144	142	Bhatia et al., 2004
5′	TcCLB.506925.300	60	60	58	63	60	Búa et al., 2001
5′	TcCLB.506563.40	110	110	110	113	110	Bartholomeu et al., 2002

*For UTRme predictions the best scoring site using T. cruzi epimastigote data is shown. UTRme 5′ enriched: UTRme predictions using In-house low pass sequencing of 5′ UTR enriched library. UTRme Li: UTRme predictions using Li et al. (2009) data. UTRme Pastro: UTRme predictions using Pastro et al. (2017) data. SLaP mapper Pastro: SLaP mapper predictions using Pastro et al. (2017) data. Exp., Experimentally defined sites. Article: Reference where the experimental prediction was described*.

Also, the availability of deep sequenced transcriptomes (Li et al., 2016) for the same *T. cruzi* stage, allowed us to check UTRme performance using reads obtained using a standard protocol RNA-seq experiment and shorter reads. As before, UTRme predictions were contrasted against the previously described UTRs. UTRme results for both approaches showed an excellent agreement with previously reported processing sites (Table 2). In most cases UTRme predicts the same UTR or a site that is within a few bases from the experimentally defined site, highlighting that the algorithm predicts sites with good precision. For those cases where the experimentally determined site was not identical to the best score site predicted by UTRme, the experimental site was usually present in the list of predicted sites with a lesser score. In the case of the deep sequenced transcriptome a greater number of processing sites was detected as reflected in the table.

To further validate our results in a genome wide scale, RNA-seq reads were simulated using randomly assigned UTRs. UTRme predicts 3′ UTRs for 7,116 genes, most of which (97.2%) are correctly assigned (within 5 nt distance of the real site). Considering multi mapping reads more genes are assigned a poly(A) site (7,884), but the accuracy diminishes significantly (91.4%). Taking into consideration the percentage of multi gene family members in the Tritryps genomes this is expected. This result prompted us not to consider multi-mapping reds by default. An analogous result is obtained for the miniexon addition site, assigning UTRs for 7,640 genes where 98.2% are correctly predicted, while when multi-mapping reads are considered the number of genes increases and a decrease in accuracy is observed (8,530 assigned 5′ UTRs with an accuracy of 92.5%).

It is interesting to note that when the dinucleotide of the 5′ splicing acceptor site is studied for the simulation, an overrepresentation of the AG dinucleotide is not observed. This is expected as UTRs lengths where randomly assigned. However, when this analysis is performed for real RNA-seq data, the AG dinucleotide is clearly the major acceptor site as expected (Supplementary Figure 2), reinforcing the accuracy of the annotations.

**Figure 4:**
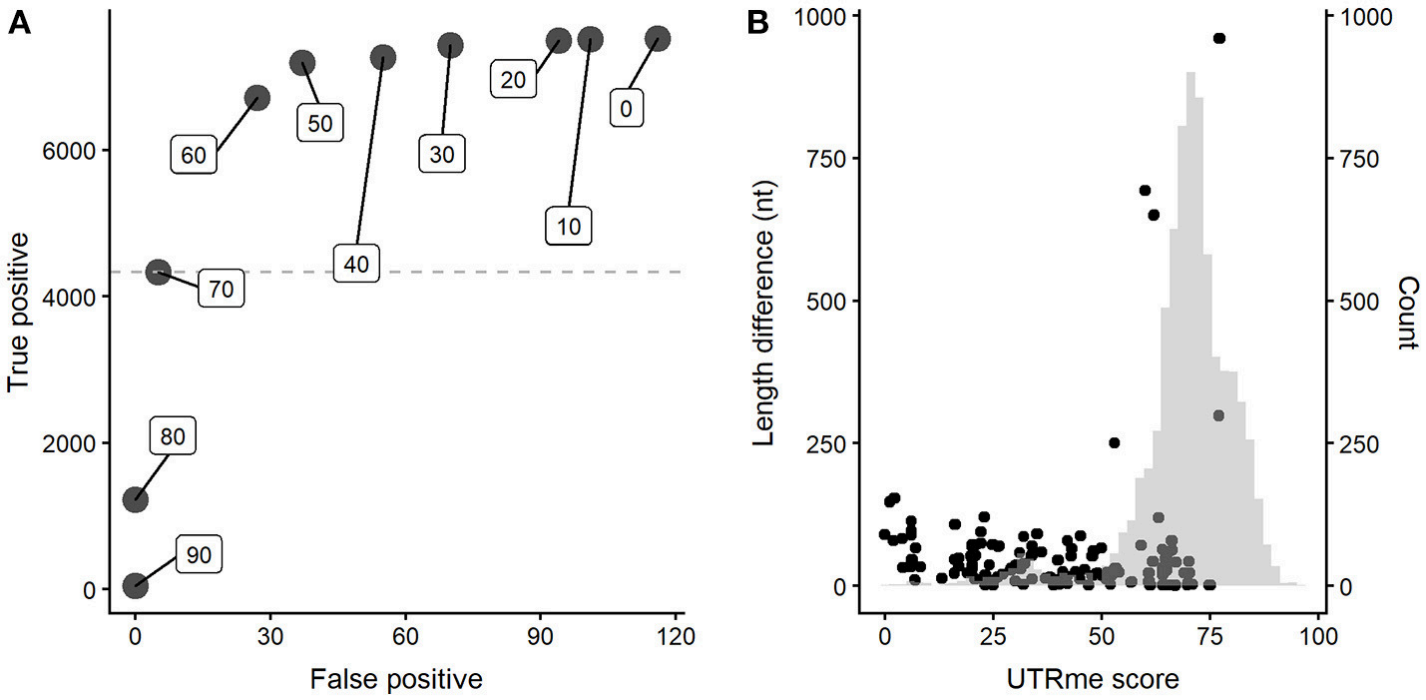
UTRme accuracy assessment for 5′ UTRs. **(A)** Dependence of the number of true positives and false positives on the UTRme score (indicated as inserts). **(B)** False positive annotations are plotted as dots indicating their score and distance to the real processing site. The histogram shows the distribution of scores for all predicted sites.

A key feature of UTRme is the reporting of a global score for each site. Positive scoring sites are given as they are supported by a reasonable amount of evidence. A higher score indicates more evidence supporting the site. Using the simulated dataset, we explored the relationship between the UTRme score and the software performance. A plot that depicts the number of correct predictions (true positives) vs. the number of incorrect assignments (false positives) for various score cutoffs was constructed (Figure 4A for the 5′ UTRs results, see Supplementary Figure 3A for the 3′ results).

The figure clearly shows that increasing the score decreases rapidly the number of false positives. High scores (>80) show virtually no false positives; as the score decreases, both the number of both true and false positives increase, but true positives increase at a higher rate. When the score reaches a value around the average, this trend starts reverting. Even though further lowering the score accomplishes an increase in true positives, this is accompanied by an increased rate of incorrect assignments. It is important to notice that the maximum number of true positives is around 7000 sites, while the maximum number of false positives is <120, even for the lowest scores. All this indicates that, as expected, incorrect assignments tend to have lower scores. This is more clearly shown in Figure 4B (and Supplementary Figure 3B for 3′ sites) where the score and distance to the real site for incorrect assignments are plotted together with a histogram representing the score for all the sites. Most false positive annotations present low scores compared to the general distribution. All this evidence supports that UTRme is a very accurate tool and that the score reflects the reliability of the predicted sites.

To test the possibility of annotating UTRs outside trypanosomes, *Echinococcus granulosus* RNA-seq data (13 paired end data from SRA Bioproject accession PRJEB5096) was examined with UTRme. The corresponding miniexon sequence was obtained from Brehm et al. (Brehm et al., 2000). One thousand eight hundred and ten sites in 1,369 genes were annotated with a 5′ UTR, while a polyadenylation site could be assigned for 6,841 genes presenting a total of 24,946 sites. These are expected results as SL addition is not pervasive in plathelminths as it is in trypanosomatids (Brehm et al., 2000). Analysis of the sequence of the trans splicing acceptor sites reveal a high percentage of the AG dinucleotide supporting the reliability of the annotated sites (Supplementary Figure 4A). A summary of UTR lengths and UTRme score distribution is shown in Supplementary Figures 4B,C.

### Comparison With Previously Available Tools

Several groups have reported tools to identify 3′ UTRs in eukaryotes, however the algorithms consider signals not clearly present in trypanosomatids and lack the possibility of studying 5′ processing sites (Xia et al., 2014; Kim et al., 2015; Grassi et al., 2016; Ha et al., 2018). For trypanosomatids, there are reports of global identification of UTRs, but in most cases the task was performed using in-house tools (Gopal et al., 2005; Siegel et al., 2005; Kolev et al., 2010; Kelly et al., 2011; Dillon et al., 2015).

**Figure 5:**
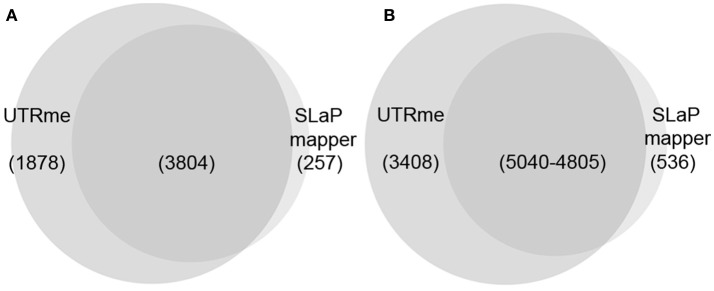
Venn diagrams comparing the results of UTRme and SLaP mapper 5′ processing sites annotations. **(A)** The intersection of the genes predicted by each tool is shown. **(B)** For genes were annotations are available for both tools, the intersection of the sites predicted by each tool is shown.

Currently, to our knowledge, the most accessible method to predict UTRs in trypanosomatid genomes is the SLaP mapper web service (Fiebig et al., 2014). To contrast UTRme results with those obtained by SLaP mapper we used 27M paired-end reads from *T. cruzi* epimastigotes (Pastro et al., 2017) (number of reads was reduced to accommodate SLaP mapper upload size limitation). In this experiment, where standard RNA-seq protocols were carried-out, UTRme was able to detect 8,448 5′ UTR regions in 5682 genes whereas SLaP mapper detected 5,343 sites in 4,061 genes. Of the genes detected by UTRme, 1878 were exclusive whereas SLaP mapper detected 257 genes exclusively. Three thousand eight hundred and four genes were detected by both software packages of which 88% had coincident predictions (Figure 5A). Of the 8,448 total sites identified by UTRme, 3,408 did not show matches with SLaP mapper, 71% were due to sites corresponding to genes detected exclusively by UTRme. SLaP mapper detected 536 exclusive sites, of which 56% were due to genes only detected by this software. The number of coincident sites is 5,040 for UTRme and 4805 for Slap mapper (the difference is due to the fact that a 5 pb window was implemented to define matching sites) (Figure 5B). The median length for the 5′ UTR regions was similar in both cases (59 and 53 bp for UTRme and SLaP mapper, respectively). While the median length for sites detected exclusively by UTRme remains around this figure (88.5), in sites detected exclusively by SLaP mapper this number increases to 786, which may be indicative of issues in these non-coincident annotations (Supplementary Figure 5A). For 3′ UTRs a similar situation was found (see Supplementary Figures 5B, 6).

**Figure 6:**
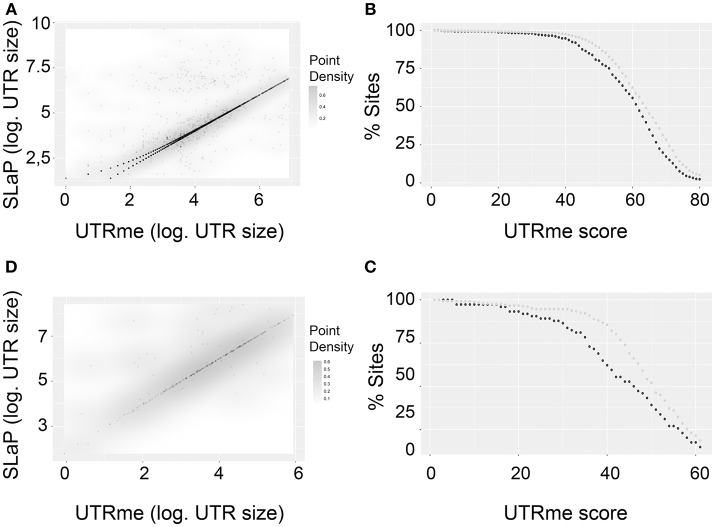
Comparison of UTRme best scoring sites with the ones predicted by Slap mapper using Pastro et al. (2017) data. **(A)** Scatter plot of 5′ UTR lengths. Darker regions indicate higher density of points. **(B)** The percentage of points that have scores above a threshold is plotted for coincident and non-coincident sites. *Dark gray:* non-coincident sites. *Light gray:* coincident sites. The percentage was calculated until the number of sites remaining is above 10 **(C,D)**. Same as **(A,B)** for 3′ UTRs.

Considering the genes where both tools predicted splicing sites, a density plot shows a very good correlation (Figures 6A,C). Interestingly, this correlation is better for sites with high UTRme score. This is shown in Figures 6B,D. Here, sites were classified as coincident if their length difference was 5nt or less or non-coincident otherwise. The percentage of coincident and non-coincident sites that are above a certain score threshold is calculated and plotted. The figure shows that this percentage decreases more rapidly for non-coincident sites than for coincident sites when the UTRme score increases. This observation supports that in cases where a high score is assigned by UTRme (which suggests that the sites can be readily identified by the reads), SLaP mapper mostly reports the same site, verifying that the score is a key factor in capturing the certainty of site definition. Nonetheless, a low score in UTRme indicates that there was less evidence to support it, which in turn likely explains the decrease in correlation with SLaP mapper predictions.

We also compared the results obtained using UTRme to analyze the RNA-seq *T. brucei* data generated by Kolev, et al. in (Kolev et al., 2010) with the ones reported by the authors. These authors constructed a SL-primed library and a 3′ end-enriched library to detect 5′ and 3′ boundaries, respectively, predicting processing sites by using an in-house pipeline. The results obtained for the comparison where similar to the ones observed for SLaP mapper (Supplementary Figures 7, 8).

Interestingly, for both comparisons UTRme was able to predict a higher number of sites. This is possibly due to the inclusion by UTRme of predictions that are discarded by other tools but that UTRme does include by penalizing them with a low score. The good correlation between the results obtained through the two tools and the influence of the UTRme score on the percentage of agreement is clearly shown in both cases.

Globally, the comparison of UTRme with available data and applications supports the software accuracy and highlights the importance and usefulness of the UTRme scores.

## Final Remarks

Post-transcriptional mechanisms are recognized as important regulatory steps in eukaryotes. Post-transcriptional mRNA regulators most commonly bind to sequences present in UTR regions, so their definition is critical to better understand regulatory networks. For trypansomatids, UTR delimiting algorithms are confounded by the presence of the A tracts in intergenic regions (Duhagon et al., 2011) and by the repetitive nature of the sequences that cause issues in the genomic assembly, among other reasons. This lead us to develop UTRme, a tool that allows not only the identification of processing sites from RNA-seq data but also reports their associated confidence. UTRme is easy to install in linux based systems, is provided with a GUI making it user friendly and it does not require previous expertise on RNA-seq data analysis, something we expect that will make the tool more readily available for wet lab biologists.

As shown by the excellent correlation with sites experimentally determined and considering the results obtained for the simulated RNA-seq data, we can conclude that UTRme predicts sites with excellent precision and that the scoring system is capable of reflecting the certainty of the annotations. The comparison with other tools allowed us to further support the advantage and usefulness of the UTRme scoring system which discriminates between sites that are clearly predicted, from those where evidence is less clear.

Finally, UTRme can be applied to predict 3′ processing sites not only in trypanosomatids but any eukaryotes and can be used for 5′ end determination in other organisms where trans splicing occurs.

## Data Availability Statement

The dataset generated for this study can be found in the SRA repository BioProject PRJNA473354.

## Author Contributions

SR and PS UTRme software development and design of the methodology. SR performed the analysis. RF and PS performed the 5′ end enriched RNAseq experiment. JS-S, BG, RF, SR, and PS wrote and reviewed the manuscript. JS-S, BG, and PS acquisition of financial support. PS coordinated the project.

### Conflict of Interest Statement

The authors declare that the research was conducted in the absence of any commercial or financial relationships that could be construed as a potential conflict of interest.
