# Supplementary material for: UTRme: A Scoring-Based Tool to Annotate Untranslated Regions in Trypanosomatid Genomes
Source: Front Genet. 2018 Dec 18;9:671. doi: 10.3389/fgene.2018.00671 (PMC6305552; doi:10.3389/fgene.2018.00671)
Supplement: Supplementary file 1 [file Data_Sheet_1.docx]

Supplementary file 1

UTRme: a scoring-based tool to annotate untranslated regions in trypanosomatid genomes

Santiago Radío^1,2^, Rafael Sebastián Fort^1,2^, Beatriz Garat^2^, José Sotelo-Silveira^1,3^, Pablo Smircich*^1,2^

## *** Correspondence:** Dr. Pablo Smircich: psmircich@fcien.edu.uy

**Description of the scoring system**

The reported score of each predicted site is obtained by combining two partial metrics: an individual and a global score. The individual score analyzes each read and its alignment to the genome independently of other reads supporting the same site, while the global score considers all reads that define a given UTR region. The sum of both defines the score of each processing site.

# Individual score

We can divide the individual scores into three metrics. Primary, secondary and accessory scores.

# Primary Score

The primary score reflects a global assessment of the accuracy of the alignment of the primary region of the read to the genome. It is calculated using a modified version of the Damerau-Levenshtein algorithm. This metric evaluates the minimum number of changes that are required to go from string A to string B considering mismatches and gaps. The higher the value reported, the smaller the distance, so there will be greater certainty in the determination of the site. If the value is under 75 the read is discarded, else the score modified according to the following table (SF1 Table). An example is shown in SF1 Fig**.**

**Table SF1**. Scores assigned by UTRme according to the Damerau-Levenshtein value calculated between the primary region of the read and the corresponding genomic sequence.

| Damerau-Levenshtein value | Primary score |
| --- | --- |
| 100 | 30 |
| 98 – 99 | 25 |
| 95 – 97 | 15 |
| 90 – 94 | 5 |
| 85 – 89 | -15 |
| 75 – 84 | -30 |
| 0 – 74 | remove |





**Fig SF1.** **(A)** the primary region is identical to the corresponding genomic region, which establishes a score of 30. **(B)** A greater divergence between these sequences is observed, obtaining a score of -15.

# Secondary score

The secondary score evaluates the secondary region and the genomic region contiguous to the primary region. UTRme discards a read if its secondary region has a Damerau‑Levenshtein value of 95 or more when compared to the corresponding genomic sequence. If the value is below 95 then a positive score of 15 is assigned. Besides, the number of mismatches is calculated (using the Hamming algorithm) and then normalized by the length of the secondary region. This ratio is multiplied by 15 and the resulting value is added to score. Besides, a positive score of 5 is assigned if it has a size greater than or equal to 15 nucleotides. Finally, the proportion of As in the secondary genomic region is calculated, multiplied by 10 and this value is subtracted from the score. See SF2 Fig for working examples.





**Fig SF2**. **(A)** When the secondary region of the read is identical to the corresponding genomic region, UTRme discards the site since it is not possible to determine with certainty the existence of a true processing site. **(B)** Considering R3 and R4 it is possible that there is a real processing site at this location. However, R1, R2, R5 probably use other poly adenylation sites as the sixth base of the secondary region (marked with a circle) has the same base as the genomic sequence (and the same between them) so it is unlikely to be a sequencing error. In this situation the predicted site of polyadenylation is not discarded but receives a heavy penalty (-20). **(C)** The predicted site of polyadenylation is discarded because the sixth base of the secondary region (marked with a circle) has in all cases the same base as the genomic sequence (and the same to each other) so that it is unlikely to be a sequencing error and therefore the site is discarded.

# Accessory scores

# Multi-mapping reads

By default multi-mapping reads are not used by UTRme to perform the annotations. If the user decides to include them in the analysis UTRme applies a negative score (-5) if the read defining a splicing site is classified as multi-mapping by bowtie2. If it corresponds to a single match read, +5 is added to the individual score.

# Putative sequencing errors in the SL or poly(A) sequence

Accounting for sequencing errors, cutadapt allows a number of differences in the search for the adapter sequences. The number of allowed mismatches is defined by the error probability parameter in cutadapt. For the SL sequence only one error is allowed. For the poly(A) the error rate is user defined (0.01 by default). The number of changes from the expected sequence is subtracted from the score.

# AG acceptor site

For the SL, if UTRme determines that the splice acceptor site corresponds to the canonical AG dinucleotide a +3 value is added, else -3 is subtracted from the score.

# Polypyrimidine tract (poly(Y))

If no tract is identified -10 is subtracted from the score. Following the poly(Y) tract characteristics described in Siegel et al 2005 the following criteria for the scores was applied: If the total length is less than or equal to 10 nucleotides, it is assigned a value of -5, else +1 is assigned. The presence of an uninterrupted poly(Y) tract greater than 15, adds +4, between 10 and 14 nt adds +2, between 6 and 9 nt adds +1. If the poly(Y) tract it is located between 10-40 nt upstream the processing site, +5 is added, otherwise -5 is subtracted. The composition of the poly(Y) tract is calculated, and positive scores are assigned to pyrimidines (+3 for each T and +2 for each C) and negative in case of purines (-2) or not determined (-5). Finally, all these scores are added, scaled to a maximum value of 100 and divided by 10 to give a maximum of 10 and a minimum of -10.

# Presence of ambiguous nucleotides (Ns)

If Ns are present in the predicted UTR region a -50 score is assigned. An option is available in the GUI for the user to discard these sites independently of the score.

# Presence of ORFs

If the UTR contains an ORF longer than a user defined threshold, the UTR is discarded. For the remaining UTRs, if ORFs longer than 90 nucleotides are present, the aminoacid sequence of the longest one is reported. In this case, a value of either -5, -10 or -50 is subtracted if this ORF is longer than 30, 50 and 100 aminoacids respectively.

# Global scores

After the individual score for each read is calculated, a global score is computed that evaluates the cumulative evidence of all the reads that support a single processing site. The factors that determine the global score of a site include the number of reads that define it (“occurrence”) and for poly(A) sites the number of non-adenine bases in the reads.

Once the number of occurrences is calculated, the score is modified according to SF2 Table.

**Table SF2.** Value added to the score according to the number of occurrences.

| Occurrence | Score |
| --- | --- |
| >= 300 | 35 |
| >= 100 | 30 |
| >= 50 | 25 |
| >= 25 | 20 |
| >= 10 | 10 |
| >= 2 | 5 |
| < 2 | 0 |

For polyadenylation sites, it is also important to consider the composition of all secondary regions. If secondary regions consist of only adenine bases the value corresponding to the number of occurrences is added to the score with a maximum of 10. If all the reads show the same non-adenine nucleotide and the number of occurrences is greater than 3, the site is discarded. If the occurrence is less than 3 or not all the reads show the non-adenine base, -10 is subtracted. When different non-adenine nucleotides are present the percentage of errors is calculated and subtracted.

# Reported score

Finally, the reported score is calculated as the global score plus the 3^rd^ quartile of the distribution of all individual scores that determine the processing site.
