# Supplementary material for: UTRme: A Scoring-Based Tool to Annotate Untranslated Regions in Trypanosomatid Genomes
Source: Front Genet. 2018 Dec 18;9:671. doi: 10.3389/fgene.2018.00671 (PMC6305552; doi:10.3389/fgene.2018.00671)
Supplement: Supplementary file 2 [file Data_Sheet_2.docx]

Supplementary Material

UTRme: a scoring-based tool to annotate untranslated regions in trypanosomatid genomes

Santiago Radío^1,2^, Rafael Sebastián Fort^1,2^, Beatriz Garat^2^, José Sotelo-Silveira^1,3^, Pablo Smircich*^1,2^

## Correspondence: *Dr. Pablo Smircich*: *psmircich*@*fcien*.edu*.uy*

# Supplementary Figures and Tables

## Supplementary Figures





**Supplementary Figure 1.** UTRs visualization. Best scoring sites predicted by UTRme visualized using the ggbio R package.

**
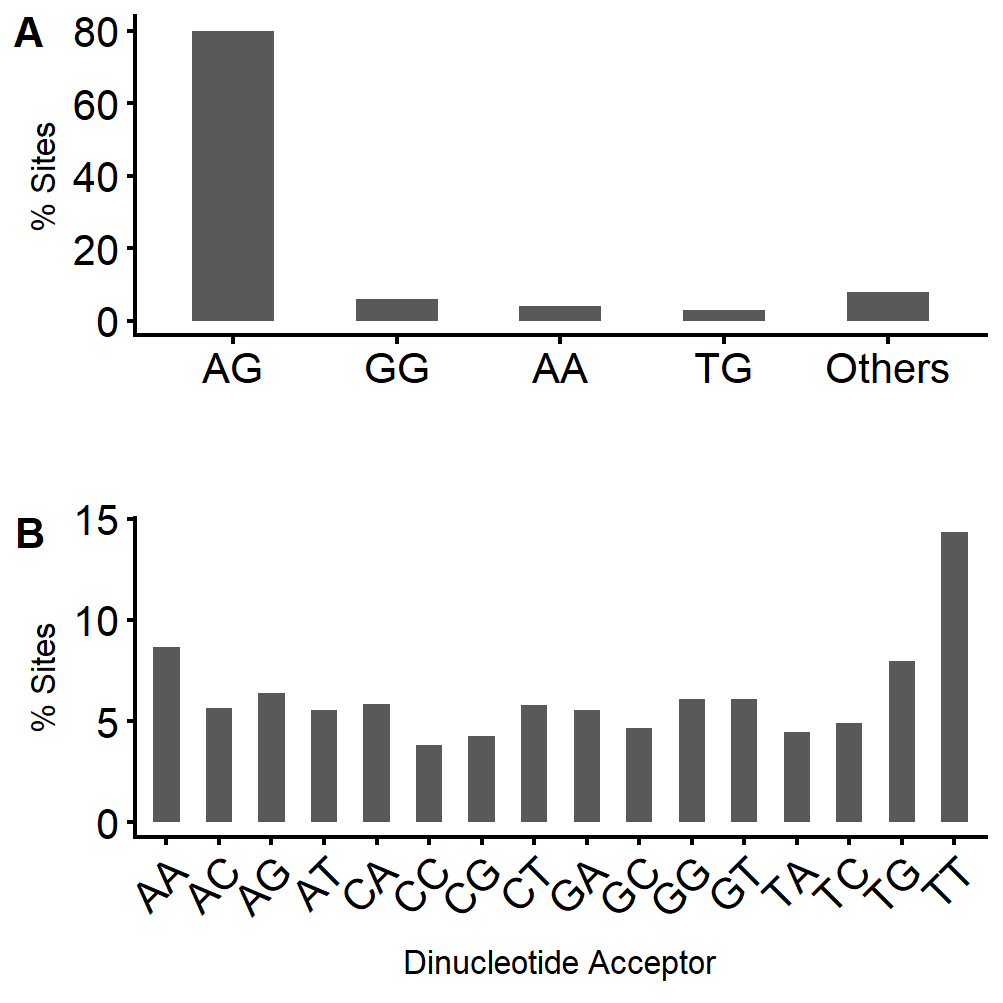
**

**Supplementary Figure 2. Frequency of dinucleotides in the 5’ acceptor site. (A)** Analysis for the simulated data set. **(B)** Analysis for the Pastro, et al. dataset.


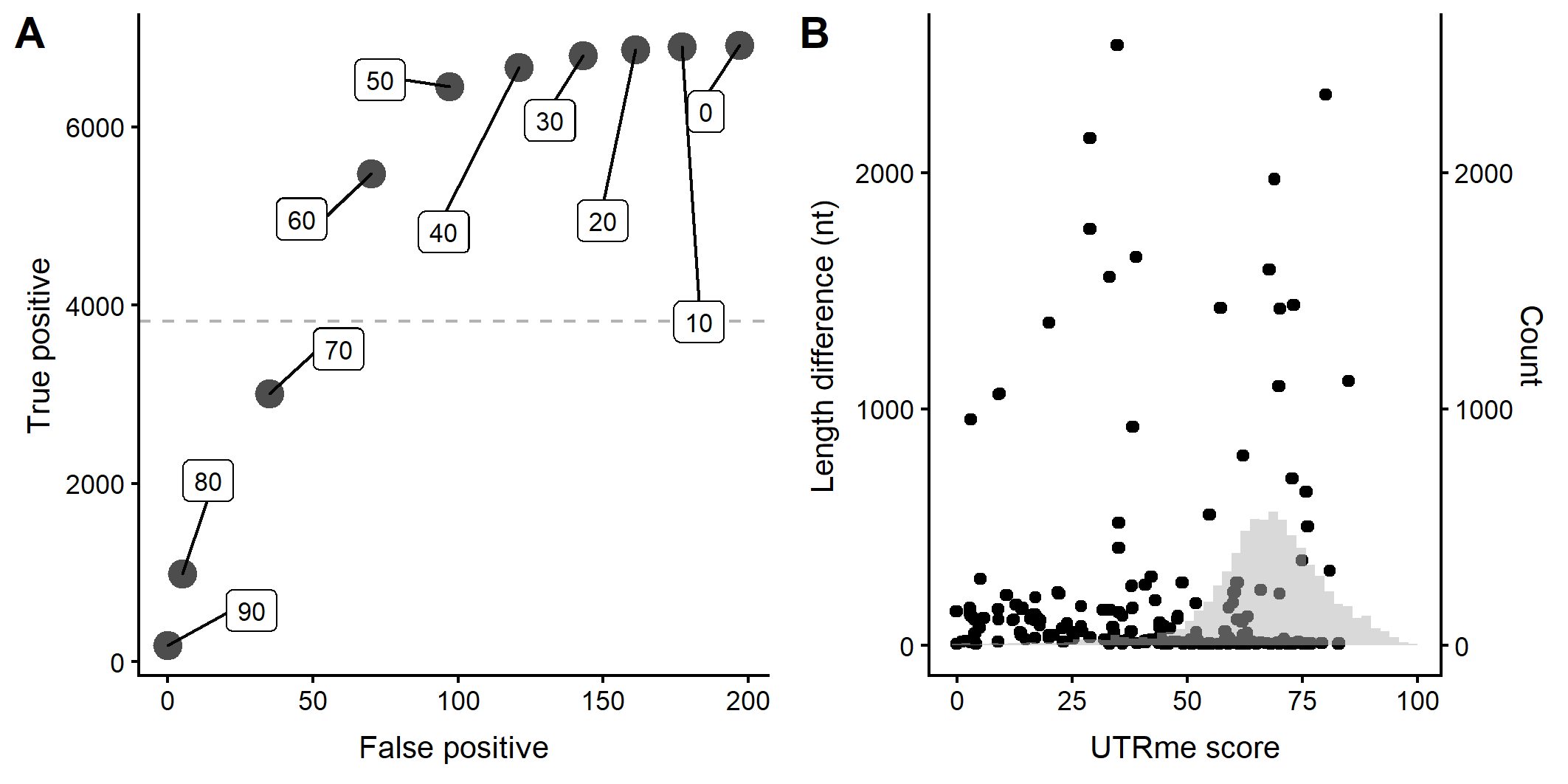


**Supplementary Figure 3. UTRme accuracy assessment for 3’ UTRs. (A)** Dependence of the number of true positives and false positives on the UTRme score (number indicated as inserts). **(B)** False positive annotations are plotted as dots indicating their score and distance to the real processing site. The histogram shows the distribution of scores for all predicted sites.

**
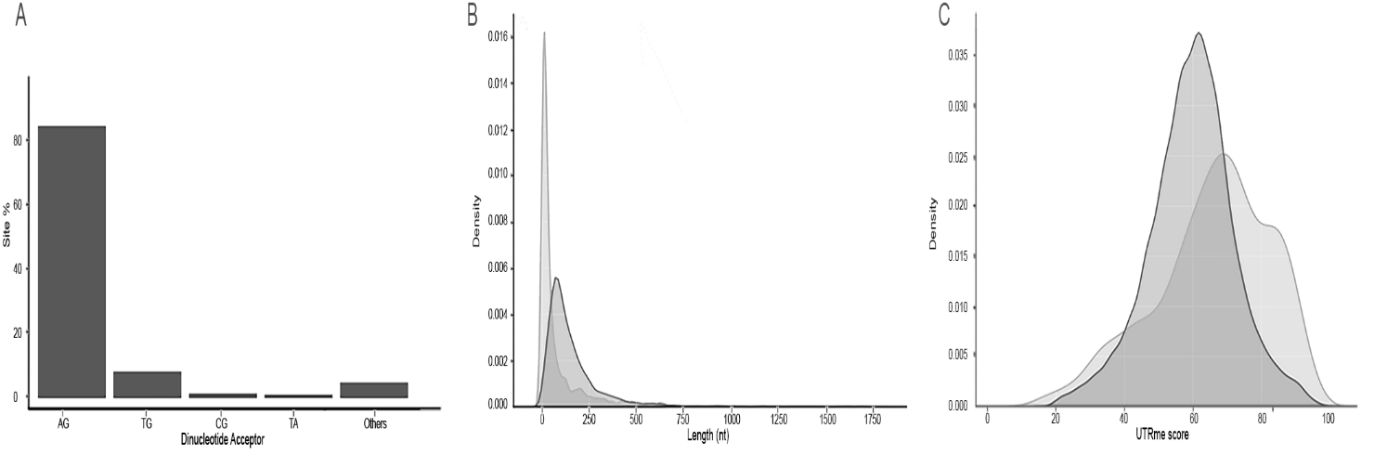
**

**Supplementary Figure 4. UTRme summary plots for the analysis of E. granulosus RNA-seq data.** Light grey: 5’ UTRs Dark grey: 3’ UTRs. **(A)** Frequency of dinucleotides in the 5’ acceptor site. **(B)** UTRs length distribution. **(C)** UTRme score distribution.

**
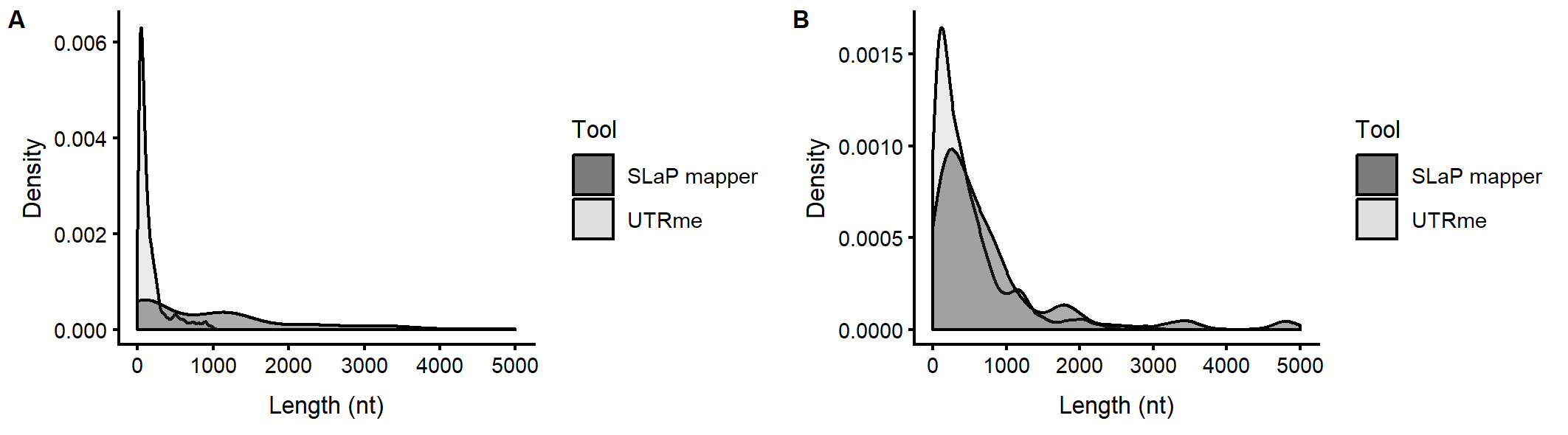
**

**Supplementary Figure 5.** Distribution of UTR non-coincident UTR lengths annotated by UTRme and SLaP mapper for the 5’ UTRs **(A)** and for the 3’ UTRs **(B)**.

**

**

**Supplementary Figure 6. Venn diagrams comparing the results of UTRme and SLaP mapper 3’ processing sites annotations. (A)** The intersection of the genes predicted by each tool is shown. **(B)** For genes were annotations are available for both tools, the intersection of the sites predicted by each tool is shown.

**

**

**Supplementary Figure 7. Venn diagrams comparing the results of UTRme and the data obtained by Kolev, et al.** The intersection of the genes predicted by each tool is shown for 5’ UTRs **(A)** and for 3’ UTRs **(B)**.

**
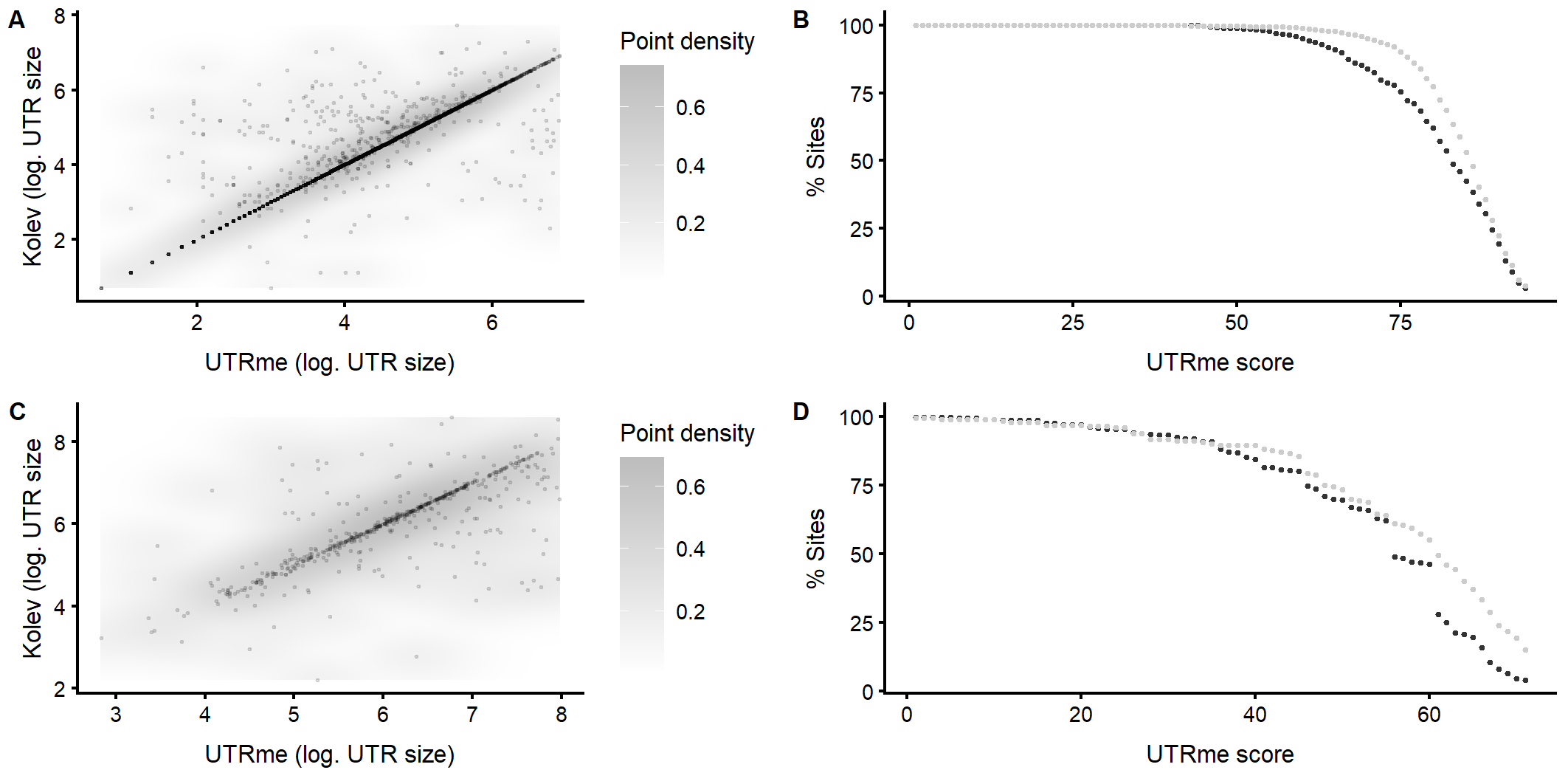
**

**Supplementary Figure 8. Comparison of UTRme output for best scoring processing sites with the ones reported by Kolev, *et al.* (Kolev et al. 2010)** **(A)** Scatter plot of 5’ UTR lengths. Darker regions indicate higher density of points**. (B)** The percentage of points that have scores above a threshold is plotted for coincident and non-coincident 5’ processing sites. Dark grey: non-coincident sites. Light grey: coincident sites. The percentage was calculated until the number of sites above the threshold is more than 10. **(C)** and **(D)** are as before for 3’ processing sites.

## Supplementary Tables

**Supplementary Table 1.** Example of UTRme full report of epimastigote's SL sites using epimastigote RNA-seq data from Li, Y., et al. (Li et al., 2016).
